# The effect of brisk walking on postural stability, bone mineral density, body weight and composition in women over 50 years with a sedentary occupation: a randomized controlled trial

**DOI:** 10.1186/s12905-016-0343-1

**Published:** 2016-09-21

**Authors:** Aleš Gába, Roman Cuberek, Zdeněk Svoboda, František Chmelík, Jana Pelclová, Michal Lehnert, Karel Frömel

**Affiliations:** Faculty of Physical Culture, Palacký University Olomouc, třída Míru 117, Olomouc, 771 11 Czech Republic

**Keywords:** Walking intervention, Pedometer, Osteoporosis, Body composition, Obesity, Falls

## Abstract

**Background:**

To assess the effect of brisk walking on postural stability, bone mineral density (BMD) and body composition in women over 50 years of age with a sedentary occupation.

**Methods:**

A 10-week walking intervention based on self-regulated brisk walking (BW) to or from work of 30–35 min at least 5 times per week. The research included a total of 104 women (58 women in intervention group). The mean center of pressure (COP) velocity in medial-lateral and anterior-posterior directions, mean total COP velocity with eyes open and closed, BMD of the distal forearm and the calcaneus, body weight, fat mass, and lean body mass were assessed.

**Results:**

The BW intervention was completed by 76 % of participants. A significant effect (time × group interaction) was confirmed only in the mean COP velocity in the anterior-posterior direction with eyes closed (*F* = 7.41*, P* = 0.008). The effect of BW was not confirmed in BMD, body weight, or body composition. The results indicate that the effect of the intervention is influenced by baseline body mass index in body weight, fat mass and visceral adipose tissue.

**Conclusions:**

BW prevents the deterioration of postural stability with eyes closed, which can have a direct effect on reducing the risk of falls under worse spatial orientation and visibility. The presented intervention model is insufficient for weight loss, changes in BMD, or body composition, and its effect should be assessed during a longer period of time.

**Trial registration:**

German Clinical Trials Register DRKS00007638, registered March 10, 2015 (retrospectively registered).

## Background

With a high prevalence and numerous related medical complications, osteoporosis is a significant health-related, economic and social issue and is especially critical due to the growing number of older individuals in the population. The most serious consequence associated with bone tissue loss is increased fragility in the area of the lumbar and thoracic spine, distal forearm and proximal femur. Serious osteoporotic fractures have a direct effect on quality of life and are associated with an increase in mortality during the first year after injury [1].

In the prevention and treatment of osteoporosis, emphasis is placed on decreasing fracture risk through interventions that lead to modifications in bone tissue metabolism and thus to an increase in bone mineral content and bone mineral density (BMD). Prevention of falls is also emphasized because falls are one of the main causes of serious osteoporotic fractures [2] and are the second leading cause of accidental or unintentional injury deaths worldwide [3]. Age- and health-adapted physical activity (PA) is one of the essential non-pharmacologic methods of prevention and treatment of osteoporosis, as evidenced by a number of studies confirming its influence on bone health [4, 5]. Additionally, PA significantly contributes to decreasing the risk of falls as a result of increased muscle strength and improved postural stability [6, 7]. Previous research indicates that overweight and obesity increases the risk of falls due to poor postural stability [8–10]. Therefore, increased habitual PA combined with weight loss is considered to be a significant factor in osteoporotic fracture prevention.

Walking is considered to be the most natural and safe form of PA and is frequently used as a specific means of PA intervention. The positive effect of short-term walking intervention on weight loss and changes in body composition has been previously confirmed [11, 12]. Weight loss improves gait parameters, walking speed and balance control [11, 13, 14], which can reduce the risk of falling and consequently risk of fractures. However, there is no clear agreement on the effect of walking on BMD and bone metabolism. Although there are studies that positively confirm the favorable effect of short- [15] and long-term walking intervention [16, 17], some authors have disproved this effect and emphasize that the results differ according to skeletal sites and recommend a combination of walking and other types of PA [18–20]. It is also known that different responses of bone tissue on walking may be genetically influenced. For example, varying effect of walking on BMD was found in relation to vitamin D receptor gene polymorphism in postmenopausal women [21].

The feasibility of walking activities included in the walking intervention program is determined by the attributes of the target group (e.g., age, health condition, type of occupation) and environmental conditions (e.g., weather and length of daylight during a specific season, safety of the location). By accepting these principles, a walking intervention model using walking to or from work can be defined. This model is well applicable in the built environment of Czech cities that are considered to be friendly to walking [22]. Aside from group attributes and environmental conditions, other factors must be considered during the planning of a walking intervention program. These include the overall length of the program and the frequency, duration, and intensity of the walking activity. The combination of these factors significantly determines participants’ adherence and overall success of the intervention program [23].

Therefore, the main objective of the study was to devise a suitable walking intervention program consisting of brisk walking (BW) to or from work and respecting the factors mentioned above and to assess the program’s effect on postural stability, BMD of the distal forearm and the calcaneus, body weight and composition in women over 50 years of age with a sedentary occupation.

## Methods

### Study sample and participant recruitment

**Fig. 1:**
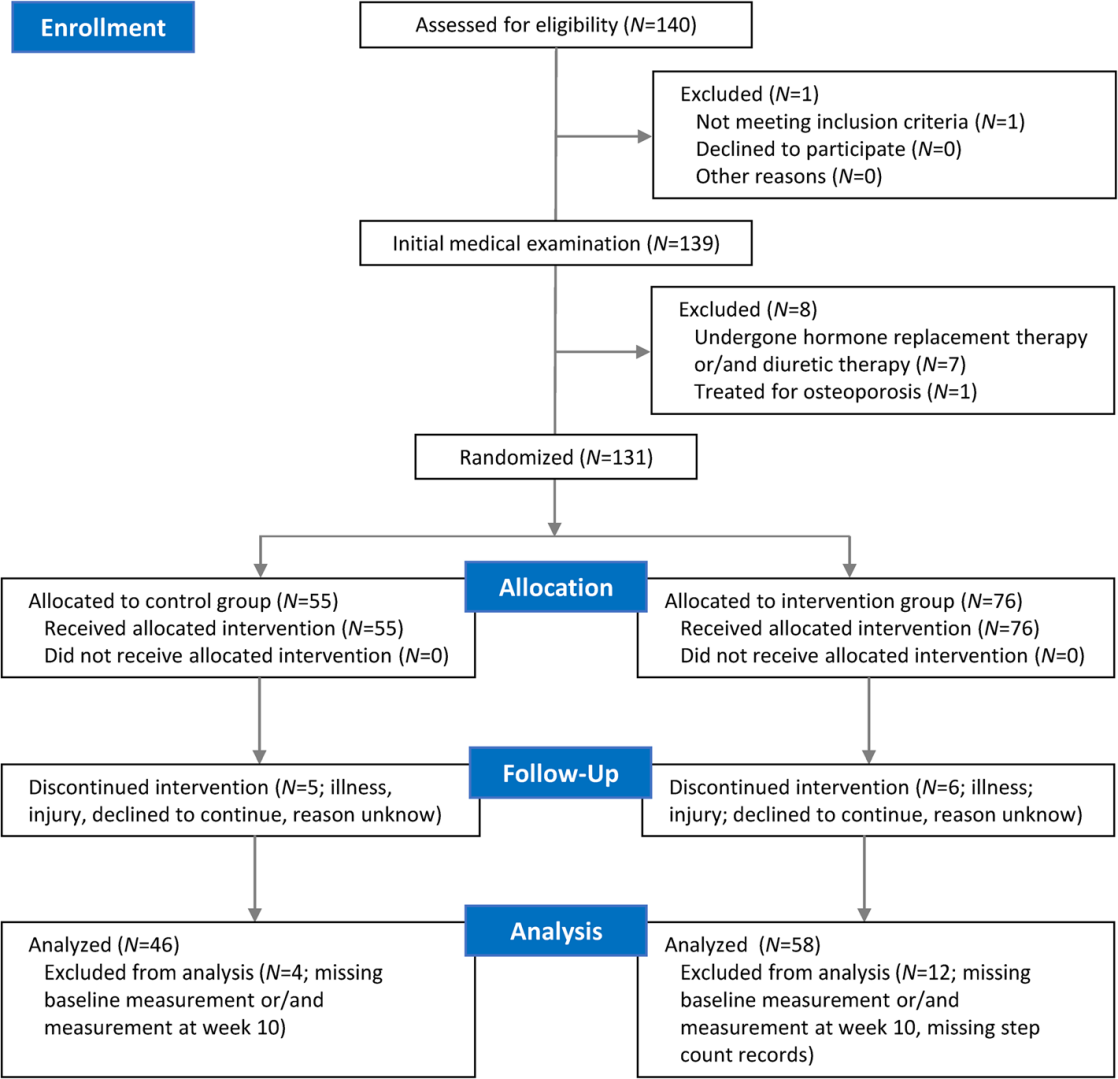
Flowchart of the participant’s recruitment, screening and assessment

The sample size was calculated based on formula by Hopkins [24]. We determined the smallest beneficial effect size 0.35, a two-tailed significance level of 1 %, statistical power of 80 %, typical error of 0.4, and a potential loss of participants of 25 %. Thus, a total of 140 women aged 50–69 years were recruited. We addressed women from institutions in Olomouc (Czech Republic), where we expected a large proportion of office workers. The recruitment strategy focused on women over 50 years of age who spend a large part of their working day seated in an office using a computer. The participants underwent an initial medical examination approximately 1 week prior to baseline measurements. Women who had had a serious fall-related fracture, had undergone hormone replacement therapy, had used diuretics during the last 24 months, had been treated for osteoporosis or had medical complications relating to the content of the intervention program (major gait, postural and neurological disorders) were excluded from the study. For ethical reasons, the women who met the exclusion criteria were not excluded from the intervention program. However, their data were not included. The research sample consisted of 131 women who were randomly divided into the intervention and control groups using simple randomization method. Random allocation was determined by the first author using a computer generated random number sequence. While processing the data, we excluded women with incomplete or invalid measurements or missing step count records (>25 % of missing values) and women who did not complete the intervention program. After taking all of these criteria into account, we obtained a sample of 104 women and analyzed their results. Figure 1 shows the flowchart of participants through the study. The basic baseline characteristics are specified in Table 1.

**Table 1:** Baseline characteristics of the study participants and intervention characteristics

	Intervention group *N* = 58	Control group *N* = 46
Age, *years*	55.9 ± 3.9	57.9 ± 5.7
Height, *cm*	163.5 ± 5.5	163.4 ± 5.0
Weight, *kg*	69.9 ± 13.0	74.3 ± 15.4
BMI, *kg/m* ^*2*^	26.2 ± 4.7	27.8 ± 5.5
Obesity^a^, *N (% of study sample*)		
Normal	26 (45 %)	14 (30 %)
Overweight	22 (38 %)	19 (41 %)
Obesity	10 (17 %)	13 (29 %)
Osteoporosis^b^, *N (% of study sample*)		
Normal BMD	25 (43 %)	16 (35 %)
Osteopenia	24 (41 %)	20 (43 %)
Osteoporosis	9 (16 %)	10 (22 %)
Baseline PA, *steps/day*	8703 ± 2847	9018 ± 3593
Meeting PA recommendation, *N (% of study sample*)		
< 5000 steps/day	5 (9 %)	6 (13 %)
5000–10,000 steps/day	36 (62 %)	26 (57 %)
> 10,000 steps/day	17 (29 %)	14 (30 %)
Length of intervention, *weeks*	9.8 ± 1.2	
PA during intervention period, *steps/day*	11,509 ± 2590	9547 ± 2976
Brisk walking intervention, *steps*	4244 ± 764	
Brisk walking/total daily steps ratio	0.39 ± 0.11	

All values are the means ± SDs

*BMI* body mass index, *BMD* bone mineral density, *PA* physical activity

^a^assessed according to baseline BMI

^b^assessed according to baseline *T*-score of the dominant forearm

### Study design

The study was performed as a controlled randomized parallel trial focusing on the assessment of the effect of a walking intervention program on postural stability, BMD, body weight and composition and was designed to meet CONSORT guidelines. This trial was retrospectively registered at The German Clinical Trials Register, identification number DRKS00007638 (registered March 10, 2015). Women in the control group were asked to not deliberately change their habitual PA and eating habits for the period of the research. The study was designed to reflect the natural integration of walking to or from work into the everyday life of the target population.

### Brisk walking intervention

The aim of the BW intervention was to increase habitual PA by incorporating BW to or from work for 30–35 min at least 5 times a week (preference of weekdays). The BW intervention took place in Olomouc where there is a relatively high index of walkability [22]. The nature of BW was individually explained to the participants by a personal assistant prior to the intervention. A BW was defined as walking at a speed that perceived breathing considerably accelerates, the body warms up and sweating occurs. Optimal walking speed was individually defined by personal assistant during the supervised walks. Another role of the assistant was to communicate with the participant on a regular basis. The assistant helped to select the most suitable walking routes in terms of safety, attractiveness, and length and to find alternative routes to increase the attractiveness of walking. This form of communication was utilized as a motivating factor to increase the participants’ adherence to the program. The intervention program was completed by 76 % of the women.

The overall length of the walking intervention was less than 1 year. Therefore, the particular season and the length of the intervention were selected to cover the longest possible homogeneous period in terms of the nature of habitual PA. We took into consideration various seasonal influences that could significantly limit the outcomes of the study. Our effort was to eliminate the effects of the different nature of weather conditions in various seasons of the year in a given region and significant deviations from a typical weekly regime in terms of PA behavior (length of daylight, holidays, and special days). Taking all of the above mentioned factors into consideration, the assumed length of the BW intervention was determined to be 12 weeks between April and June. However, the real conditions limited the possibility of performing the intervention within the intended length, so the average length of the intervention was 9.8 ± 1.2 weeks (Table 1).

### PA monitoring

The volume of walking was expressed by means of step counts recorded by a Yamax DigiWalker 700 SW instrument (Yamax Co., Yasama Corp., Tokyo, Japan). The participants wore the pedometer on their right side level with the center of gravity of the body close to the iliac crest every day throughout the intervention period. The pedometer was reset and attached immediately after the participants woke up and was removed before going to bed. During the day, the participants used specially designed record sheets to record step counts at the beginning and at the end of BW sessions and in the evening before the device was removed.

### Postural stability

Postural stability during a 30 s stance was recorded with eyes opened and closed using a Kistler force plate (type: 9286 AA, Kistler Instrumente, Wintherthur, Switzerland). To assess postural stability, the following three postural sway parameters were used: the mean total center of pressure (COP) velocity and mean COP velocities in the medial-lateral and anterior-posterior directions. The results of two trials for each condition (order was random) were averaged. The data were filtered using a fourth order low-pass Butterworth filter with a cut-off frequency of 7 Hz using MATLAB version R2010b (Mathworks Inc., Natick, MA, USA).

### Bone tissue measurement

BMD was measured on the right and left distal forearm and the calcaneus using a peripheral densitometer EXA-3000 (Osteosys, Seoul, Korea) with digital radio beam pDEXA (0.1 mSv). The device calibration was always performed in the morning and when the device was idle for over 2 h using factory QC phantom (precision error <2 % in vivo). The measurement was performed in a laboratory under the supervision of experienced radiology technologists. The measurement was performed in a sitting position, the calcaneus was bare, and all metal objects (watch, jewelry, etc.) were removed from the distal forearm. The regions of interest were selected automatically using the manufacturer’s software. Osteoporosis prevalence was evaluated using the World Health Organization recommendation [25] relating to *T*-score values on the dominant limb.

### Body composition and anthropometric indicators

Body composition was assessed by a multi-frequency bioelectrical impedance analysis with the manufacturer’s equation. Body composition assessment using the InBody 720 device (Biospace Co., Ltd., Seoul, Korea) is sufficiently valid for the target age group [26]. The total impedance was measured using 6 frequencies, from 1 to 1000 kHz, and the reactance to mean frequencies of 5 to 250 kHz. The participants were instructed in advance on recommendations to observe for a period starting 48 h before the measurement to maintain examination validity.

Body height was measured prior to body composition assessment using a digital stadiometer BSM 370 (Biospace Co., Ltd., Seoul, Korea) with an accuracy of 0.1 cm. Body weight was measured to the nearest 0.1 kg with an InBody 720 device. Body mass index (BMI) was calculated by dividing body weight (kg) by body height squared (m^2^). Body height, body weight and BMI were considered as secondary outcomes measures.

### Statistical analysis

To assess the effect of BW on postural stability, BMD, body weight and composition, two-way repeated measures analysis of variance (ANOVA) was used. We monitored the effect of the time factor (2 levels with repeated measures), effect of the group factor (2 levels) and total effect (time × group interaction). The significance of the differences between the baseline measurement and measurement in week 10, and the differences between the intervention and control group at baseline and in week 10 were analyzed for individual variables after two-way repeated measures ANOVA using Fisher’s LSD post-hoc test. Pearson’s *r* was used to assess the relationship between a percentage change from the baseline measurement in the monitored variables and the baseline BMI and PA. One-way ANOVA was used to assess the differences in the basic characteristics of the sample at the beginning of the BW intervention. Regarding the rough nature of the data, high variability of the intervention factor and size of the sample, a level of statistical significance of 1 % (*P* < 0.01) was determined for all of the statistical analyses.

## Results

One hundred and four women were included in the study. Sixty-one percent of them were postmenopausal with mean age of menopause 51.5 ± 3.2 years and 4.7 ± 5.7 years since menopause. The baseline characteristics of age and anthropometric figures and baseline PA did not differ between the intervention and control groups (Table 1). A higher number of individuals who were overweight and obese was observed in the control group, where 70 % of participants reported BMI values >25 kg/m^2^. The control group included a higher number of women suffering from osteoporosis diagnosed according to the *T*-score in the distal forearm of their dominant limb. The data on baseline PA indicated that approximately one-third of the participants had performed over 10,000 steps/day prior to the intervention. Participants accumulated 4244 ± 764 steps per average BW session which corresponds to a step cadence ranging from 142 to 121 steps/min for BW session in duration of 30 to 35 min.

The baseline values of postural stability, BMD, body weight and composition did not differ between the intervention and control groups (Table 2). During the monitored period, the control group recorded an increase in the mean total COP velocity and an increase in the mean COP velocity in the medial-lateral and anterior-posterior direction with eyes closed from 12.1 to 30.1 %. The intervention group recorded a decrease in total COP velocity with eyes opened about 7.9 %. The total effect (time × group interaction) of BW was confirmed only in the mean COP velocity in the anterior-posterior direction with eyes closed (*F* = 7.41*, P* = 0.008).

**Table 2:** Change in postural stability, bone mineral density, body weight and composition after a brisk walking intervention

	Intervention group *N* = 58		∆	%∆	Control group *N* = 46		∆	%∆	*F*_(1, 102)_	*P*-value
	Baseline	Week 10			Baseline	Week 10				
Postural stability										
V_EO_, *mm/s*	11.4 ± 3.2	10.5 ± 2.9	–0.9*	–7.9 %	11.6 ± 2.6	11.5 ± 2.7	–0.1	–0.9 %	2.73	0.102
V_EC_, *mm/s*	14.4 ± 4.9	14.1 ± 5.6**	–0.3	–2.1 %	14.9 ± 4.3	18.2 ± 8.9	3.3*	18.1 %	6.79	0.011
Vx_EO_, *mm/s*	5.2 ± 1.8	4.6 ± 2.1	–0.6	–11.5 %	4.6 ± 1.5	4.7 ± 2.0	0.1	2.1 %	3.32	0.071
Vx_EC_, *mm/s*	6.5 ± 2.9	6.4 ± 4.3	–0.1	–1.5 %	5.8 ± 2.7	8.3 ± 6.3	2.5*	30.1 %	4.61	0.034
Vy_EO_, *mm/s*	9.0 ± 2.7	8.4 ± 2.3	–0.6	–6.7 %	9.6 ± 2.5	9.4 ± 2.3	–0.2	–2.1 %	1.07	0.303
Vy_EC_, *mm/s*	11.4 ± 4.0	11.0 ± 3.7**	–0.4	–3.5 %	12.4 ± 3.7	14.1 ± 5.7	1.7*	12.1 %	7.41	0.008
Bone mineral density										
Right forearm, *g/cm* ^*2*^	0.418 ± 0.074	0.416 ± 0.072	–0.002	–0.5 %	0.390 ± 0.070	0.389 ± 0.069	–0.001	–0.3 %	0.02	0.883
Left forearm, *g/cm* ^*2*^	0.392 ± 0.068	0.387 ± 0.063	–0.005	–1.3 %	0.373 ± 0.066	0.366 ± 0.068	–0.007	–1.9 %	0.10	0.759
Right calcaneus, *g/cm* ^*2*^	0.494 ± 0.108	0.497 ± 0.108	0.003	0.6 %	0.527 ± 0.110	0.518 ± 0.104	–0.009	–1.7 %	6.16	0.015
Left calcaneus, *g/cm* ^*2*^	0.496 ± 0.103	0.507 ± 0.107	0.011	2.2 %	0.512 ± 0.099	0.516 ± 0.096	0.004	0.8 %	1.08	0.302
Body weight and composition										
Body weight, *kg*	69.9 ± 13.0	69.4 ± 12.5	–0.5	–0.7 %	74.3 ± 15.4	73.9 ± 15.1	–0.4	–0.5 %	0.37	0.543
Fat mass, *kg*	24.5 ± 9.3	23.7 ± 8.6	–0.8*	–3.3 %	27.7 ± 11.5	27.0 ± 11.1	–0.7*	–2.6 %	0.19	0.656
Fat mass, *%*	34.0 ± 7.1	33.2 ± 6.7	–0.8*	–2.4 %	35.9 ± 8.1	35.2 ± 7.9	–0.7*	–2.0 %	0.03	0.859
Visceral fat area, *cm* ^*2*^	109.7 ± 35.6	105.2 ± 32.8	–4.5*	–4.1 %	120.4 ± 40.5	116.6 ± 39.6	–3.8*	–3.3 %	0.36	0.553
Lean body mass, upper limbs, *kg*	4.6 ± 0.8	4.6 ± 0.8	0.0	0 %	4.9 ± 0.8	4.9 ± 0.8	0.0	0 %	1.47	0.229
Lean body mass, trunk, *kg*	20.3 ± 2.6	20.2 ± 2.5	–0.1	–0.5 %	21.0 ± 2.4	21.0 ± 2.4	0.0	0 %	1.61	0.207
Lean body mass, lower limbs, *kg*	13.9 ± 1.8	14.0 ± 1.8	0.1*	0.7 %	14.2 ± 1.9	14.4 ± 2.0	0.2	1.4 %	0.01	0.965

All values are the means ± SDs

*V* mean total COP velocity, *Vx* mean COP velocity in the medial-lateral direction, *Vy* mean COP velocity in the anterior-posterior direction, *EO* eyes opened, *EC* eyes closed

∆ difference between week 10 and baseline

%∆ percentage difference between week 10 and baseline

*P < 0.01, effect of intervention; LSD post-hoc test after two-way repeated measures ANOVA

**P < 0.01 difference between intervention and control group; LSD post-hoc test after two-way repeated measures ANOVA

A significant total effect of BW on BMD of the distal forearm and calcaneus and body weight was not confirmed. We observed statistically significant differences in the variables relating to fat tissue in the intervention group. We confirmed a significant loss of fat mass (FM) by 0.8 kg (*P* < 0.001), loss of FM percentage by 0.8 % (*P* < 0.001) and loss of visceral fat area by 4.5 cm^2^ (*P* < 0.001). We also observed an increase in lean body mass in the lower limbs by 0.1 kg (*P* = 0.005). Although we observed statistically significant changes in body composition, these changes cannot be attributed to BW regarding to corresponding changes in the control group.

**Fig. 2:**
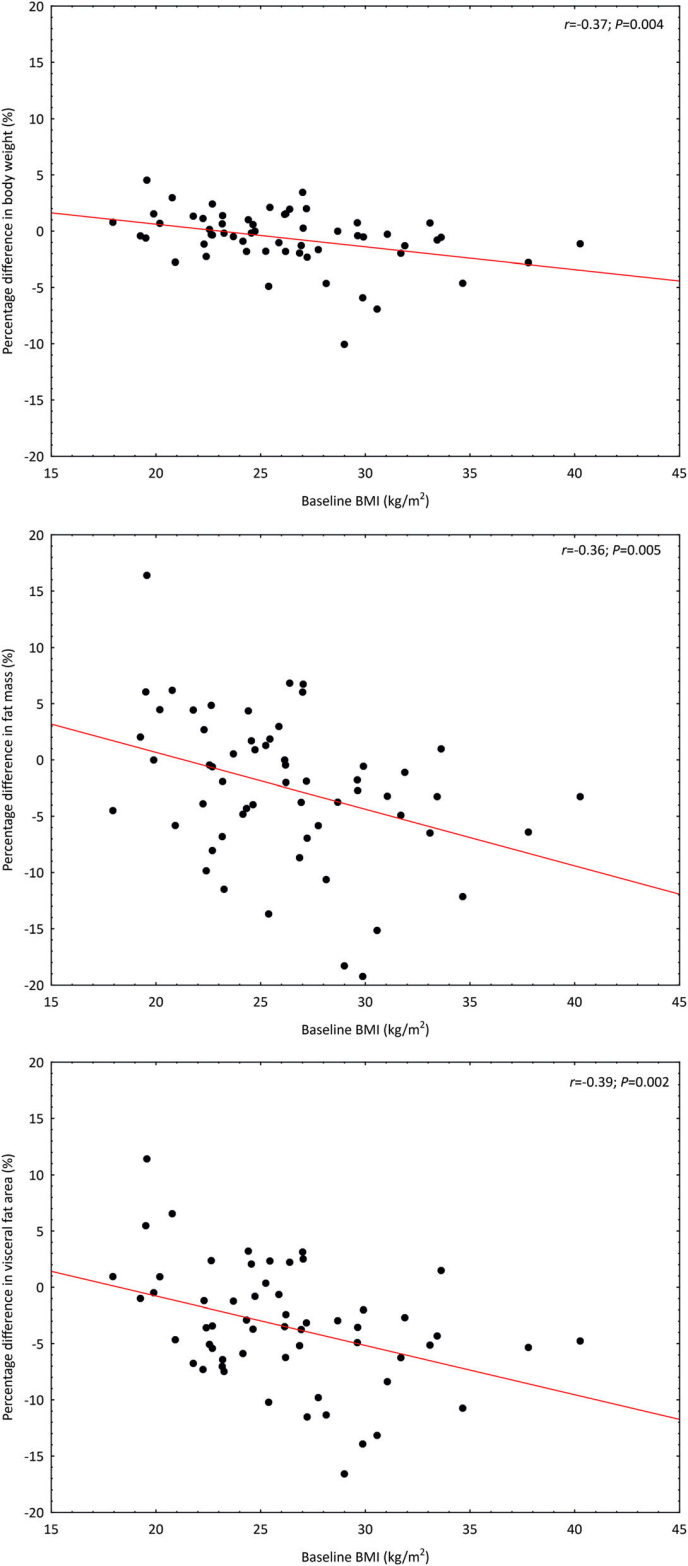
Relationship between the percentage difference from the baseline in body weight, fat mass, and visceral fat area and baseline body mass index in the intervention group (*N* = 58). *Note*: BMI – body mass index

Regarding the fact that the success of the BW intervention can be affected by baseline BMI and PA, we assessed the result of BW (percentage change from baseline) with respect to these parameters. A negative linear correlation was confirmed between BMI and the percent change in body weight, FM, and visceral adipose tissue (Fig. 2). A significant correlation between baseline PA and the percent change in the monitored parameters of postural stability, BMD, body weight and composition was not confirmed.

## Discussion

The positive effect of PA interventions was previously confirmed in connection with the majority of non-communicable diseases. At the same time, PA interventions are a suitable means of decreasing the risk of falls [6]. However, the success of PA interventions and participants’ adherence is primarily affected by the length of the intervention program, type of PA intervention and environments where the intervention is performed. The proposed design of the study indicates successful adherence of women to BW intervention, as only 6 women of the intervention group withdrew during the intervention. The demands for the research participants included not only the intervention itself but also activities associated with data collection and recording. In this case, the participants had to record step counts on a daily basis.

Currently, there is no study that assesses the direct effect of BW on postural stability in individuals with sedentary occupations. In our study, the intervention group recorded significant improvements in postural stability in a standing position with eyes opened, while the control group achieved a significant increase in the total COP velocity and COP velocity in the medial-lateral and anterior-posterior direction with eyes closed. These results support the interpretation that BW contributed to improved balance in the area of visual control and spatial orientation (eyes opened) and to maintaining the level of balance in the area of vestibular and somatosensory systems (eyes closed). However, the total effect of BW was confirmed only in the mean COP velocity in the anterior-posterior direction with eyes closed. A post-hoc analysis indicates that this is caused by a significant increase in COP velocity in the control group, while no changes after the baseline measurement were observed in the intervention group. Although 10 weeks of BW does not lead to a significant improvement in postural stability, it has a significant positive effect of preventing deterioration in postural stability in the anterior-posterior direction under worse visibility and thus can contribute to a decreased risk of falls. According to Kurz et al. [27], the deterioration of anterior-posterior postural control is associated with a higher risk of serious injury following fall events. A prospective study by Brauer et al. [28] showed that alone measures of COP motion in a quiet stance had a poor ability to predict individuals who would fall, but had a good ability to identify most individuals who would not fall. In this context, a lower COP velocity in the intervention group indicates that a higher proportion of these women are not at risk of falling.

PA intervention is one of few intervention models that promotes bone health and increases the level of muscle strength simultaneously, which are correlated to a reduced risk of falls. On the other hand, there is evidence that although BW intervention has clinically important impact on BMD, it is also associated with an increased risk of falls [29]. This issue should be taken into account when BW is advised for subjects with poor postural stability.

The effect of PA on BMD is site-specific and was previously confirmed in the proximal femur, lumbar spine and the calcaneus [16, 18, 19, 30–32]. However, its effect in the forearm was weak [19, 33]. The nature of the intervention that PA belongs to is an important factor that might influence the effect of the intervention program. While the American College of Sports Medicine recommends a combination of weight-bearing endurance activities and resistance exercises to ensure bone health [34], there are studies that assess the effect of walking as a singular exercise therapy (walking-only intervention program) on bone health.

While the results of cross-sectional studies point to an association between step counts per day and BMD in various skeletal sites [4, 5], the effect of walking-only interventions on BMD is not entirely clear and varies according to skeletal sites [18–20]. Contrary to other parts of the skeleton, the calcaneus is subjected to repeated loads and a relatively high ground-reaction force during walking, which increases with increasing speed [35, 36]. This indicates that the anticipated effect of walking could have a positive effect on this part of the skeleton and even intensified after the application of BW with average speed higher than usual walking in adults and older adults [37]. Moreover, Boyer et al. [5] claim that the influence of walking on BMD is affected not only by speed but also by the body weight of an individual. To achieve the same effect, women with a lower body weight must accumulate more steps than women with a higher body weight or must perform the same amount of steps at a higher speed. In this respect, it appears that BW interventions provide a higher potential than walking interventions, where the main goal is to accumulate a certain amount of steps per day (usually 10,000 steps/day) irrespective of walking speed.

The anticipated positive effect of BW on BMD of the distal forearm and calcaneus was not confirmed in this study. This could be explained by the length of the BW intervention. As a result of all of the aspects presented in the Methods section of this paper, the length of BW intervention was close to a period of 10 weeks. Although Yoo et al. [15] found the positive effect of 3-month walking intervention on bone metabolism, we did not observed any significant changes for distal forearm nor for calcaneus BMD. It is possible to assume that length of intervention is the main reason of this result. According to Kohrt et al. [34], a period of at least 6 months is required for measurable new steady-state bone mass changes. A positive effect of BW on BMD of the calcaneus is described by Brooke-Wavell et al. [16] after a 12-month BW intervention in 84 postmenopausal woman. A positive effect of walking interventions exceeding 6 months on proximal femur BMD was demonstrated in a meta-analysis study by Ma et al. [19]. However, as mentioned in the Methods section, experimental verification of the effect of BW for a period longer than 6 months is impossible due to seasonal variations and the amount and nature of PA.

While Murphy and Hardman [12] observed a significant decrease in the initial body weight as well as FM in adult women engaged in BW of identical length (i.e., 10 weeks) and frequency (i.e., 5 days per week), our results did not confirm this effect. The results indicated a weight loss of 1 % and a decrease in the FM, FM percentage and visceral fat area of 3.3 %, 2.4 %, and 4.5 %, respectively. In the study, the frequency and length of BW was selected in a way that the total amount of PA corresponds with acknowledged recommendations of approximately 150 min/week (burning approximately 1000 kcal/week). Based on our findings, we assume that the determined length and level of the intervention is not sufficient and that the success of BW intervention would theoretically improve after applying a higher amount of PA. This is also confirmed by Jeffery et al. [38], who discovered that higher levels of PA (burning 2500 kcal/week) promote long-term weight loss better than conventional recommendations of burning 1000 kcal/week. It should be noted, however, that an increasing amount of intervention PA causes decreased adherence to a walking prescription. Schutz et al. [39] recorded an excellent adherence to a walking prescription of 30 min 5 times per week in normal weight and overweight women, while observing a significant decrease in adherence after an increase to 60 and 90 min 5 times per week.

Donnelly et al. [40] claim that the crucial factor determining the effectiveness of PA intervention is the duration of intervention PA rather than frequency. The authors investigated moderately obese females who performed 30 min of continuous PA 3 days/week and observed a significantly higher weight loss compared with participants who performed 150 min/week of BW (two 15 min sessions 5 days/week). Apart from the duration and frequency, BW is also determined by intensity. Studies include various approaches. In some studies, BW intensity was specified as specific percentages of heart rate [11, 12]. In this study the intensity was self-regulated which could influence the efficacy of a BW intervention, especially if the intensity of BW would be lower than we requested. However, our results provide evidence that intensity of BW was such as reported in Compendium of Physical Activities [41]. The intensity of BW was estimated to range from 4.5 to 6.8 METs and was calculated using equation for determination of metabolic equivalent of walking from steps cadence [42].

The effectiveness of a BW intervention is influenced by the baseline characteristics of the participants. In this study, the main inclusion criteria were age and type of occupation. Therefore, the overall analysis included women with a relatively wide range of BMIs (17.9–40.3 kg/m^2^). For this reason, in our analysis of the effect of BW, we monitored the influence of baseline BMI on postural stability, BMD, body weight and composition parameters. We confirmed a negative linear correlation between the BMI and percentage change in body weight, FM and visceral adipose tissue. A vast majority of women in the intervention group maintained their baseline body weight, with only 3 women experiencing a change exceeding 5 %. As far as FM is concerned, we observed more significant individual changes, ranging from –19.2 to 16.4 %. Sixteen women reduced their FM by more than 5 %, which is considered to be a significant change from a clinical perspective [43], especially in women with a BMI >25 kg/m^2^. On the contrary, in normal weight women, no significant changes in body weight or FM were observed. This leads to a conclusion that in these women, the given intervention program had a predominantly preventive nature. In this context, BW appears effective in the sense of reducing FM in women with a higher BMI despite the length of the intervention program.

## Conclusions

BW led to a maintained level of postural stability when the participants’ eyes were closed. The effect of BW on postural stability with eyes opened, BMD, body weight and composition was modest. However, analysis indicated that the effect of BW on body weight and body composition was influenced by baseline BMI. A significant reduction of FM was observed in participants with BMI >25 kg/m^2^, while in normal weight women the selected intervention model was of a protective nature. Participants’ adherence rates indicated that the selected intervention model was well accepted and seems to be well implemented in the daily regime. However, additional research should verify the long-term effectiveness of BW and assess its effect on a wider range of parameters that directly influence the risk of fall-related osteoporotic fractures.

## Abbreviations

ANOVA: Analysis of variance
BMD: Bone mineral density
BMI: Body mass index
BW: Brisk walking
COP: Center of pressure
FM: Fat mass
PA: Physical activity
